# A Study of the Influence of Ion-Ozonized Water on the Properties of Pasta Dough Made from Wheat Flour and Pumpkin Powder

**DOI:** 10.3390/foods13203253

**Published:** 2024-10-13

**Authors:** Bauyrzhan Iztayev, Auyelbek Iztayev, Talgat Kulazhanov, Galiya Iskakova, Madina Yakiyayeva, Bayan Muldabekova, Meruyet Baiysbayeva, Sholpan Tursunbayeva

**Affiliations:** Faculty of Food Technology, Almaty Technological University, Almaty 050012, Kazakhstan; niipt@atu.kz (B.I.); a.iztaev@atu.kz (A.I.); tkulazhanov_atu@mail.ru (T.K.); yamadina88@mail.ru (M.Y.); bayan_10.04@mail.ru (B.M.); meruyet-80@rambler.ru (M.B.); sh-tursunbayeva@mail.ru (S.T.)

**Keywords:** whole-grain flour, pumpkin powder, ion-ozonated water, pasta, technological modes, quality

## Abstract

Water treated with ion ozone improves the technological qualities of food products. Therefore, ion-ozonated water was used in the work, and whole-grain flour from soft wheat of the Almaly variety and pumpkin powder were used as raw materials to improve the quality and nutritional value of the pasta. This study investigated the effects of ion-ozone concentration in ion-ozonated water C_io_, water temperature t_w_, pumpkin powder content C_pp_ and drying temperature t_d_ on various characteristics affecting the quality of pasta, including its organoleptic physical, chemical, and rheological properties. These characteristics were assessed by conducting multiple experiments, a total of 25 indicators were determined, such as humidity, acidity, cooking properties, deformation, and other basic quality indicators. To reduce the number of experiments and obtain a reliable assessment of the influence of individual factors on the quality indicators of pasta, methods involving the multifactorial design of experiments were applied. Data processing and all necessary calculations were carried out using the PLAN sequential regression analysis program. Consequently, our findings indicate that minimizing dry water (DM) loss in cooking water requires a dual approach: increasing ion-ozone concentration and optimizing pasta composition and drying conditions, specifically by reducing pumpkin powder content and drying temperature. As a result, it was established that to obtain high-quality pasta from whole-grain flour with high quality and rheological properties, it is necessary to use the following optimal production modes: ion-ozone concentration in ion-ozonated water C_io_ = 2.5 × 10^−6^ mg/cm^3^, water temperature t_w_ = 50 °C, pumpkin powder content C_pp_ = 3.0%, and pasta drying temperature t_d_ = 50 °C. The resulting pasta is an environmentally friendly product with a high content of biologically active substances.

## 1. Introduction

In recent years, with the well-being of the population becoming a growing concern and the development of the global information field, the need for healthy and individualized nutrition has emerged in society. Developing and producing next-generation food products for healthy, functional and therapeutic nutrition is an innovative direction in the food industry, which is of paramount practical importance, promoting social efficiency. To create a new assortment of such products, there is an increased and steady trend of growing consumer interest in food products enriched with natural biologically active substances, including those of plant origin. This is attributed to their widespread availability and renewability, and the consumer preferences of diverse demographic groups. Food additives are used in various food transformation processes, and food safety has become a major concern for consumers [1,2]. The rich plant resources of Kazakhstan open up enormous prospects and broad opportunities for researchers in terms of searching for new biologically active substances and creating new practically valuable food products based on them [3,4].

Unlike high-grade flour, whole-milled flour retains all the components of the wheat grain [5,6]. In whole-wheat flour, all the anatomical components of the grain, such as the endosperm, germ, and hull layers, are present in the same proportions as in the grain. This flour contains significantly more dietary fiber, vitamins, and minerals compared to high-quality flour [7]. Whole-grain wheat flour is a product that is obtained through a single grinding of cereal grains, without further sifting to separate grain particles according to their quality and size; that is, the wheat flour is used entirely. This flour contains coarse particles ranging in size from 0.5 to 1.5 mm [8].

Thus, whole-grain flour is a storehouse of vitamins, microelements, proteins, and carbohydrates that provide energy, as well as polyunsaturated fatty acids that have a positive effect on metabolic processes in the body. Whole-grain consumption has been consistently associated with improved cardiovascular disease outcomes and a healthy lifestyle in large observational studies. High-fiber whole grains (oats, barley) reduce serum low-density lipoprotein cholesterol and blood pressure, and also improve the response to glucose and insulin [9,10].

The consumption of whole grains leads to weight loss through multiple mechanisms, given the lower energy density of whole-grain-based foods, lower glycemic index, the fermentation of indigestible carbohydrates (satiety signals), and finally, the modulation of gut microflora [11]. The consumption of whole grains is associated with a lower risk of ischemic stroke [12,13].

Pasta products are constantly in great demand among residents of many countries, so they are a promising object for the enrichment and use of non-traditional raw materials for the production of pasta [14,15,16]. Consumers opt for it due to its inexpensive cost and low glycemic index, as well as its ease of processing, preparation, storage and transportation.

An effective method to enhance the nutritional value of grain products (for example, pasta) is to create multigrain mixtures from whole-grain flour, balanced in amino acid, mineral and vitamin composition [17,18]. Studies [19,20] have established that the addition of whole-grain flour (20%) replacing part of the wheat flour has a beneficial effect on the quality composition of the finished product.

Fresh pasta (FP) was prepared by mixing semolina with a liquid starter and semolina-based whole-grain semolina, and the effect of inclusion of the starter was assessed in comparison with a control sample (CP) prepared using semolina and whole-grain semolina. In general, FP samples were characterized by improved nutraceutical properties, namely a higher content of free essential amino acids and phenolic compounds, a lower content of phytic acid, and higher antioxidant activity [21,22]. In [23], to improve the taste and nutritional properties of fresh pasta, prevent lipid oxidation, and increase shelf life, a starter obtained from wheat germ and whole-grain semolina was used. Three different formulations were prepared: the first using semolina, the second using raw wheat germ, whole-grain semolina, and semolina, and the last combined semolina and sourdough.

To expand the range of therapeutic and preventive nutrition products, new technology to make pasta from spelled flour and vegetable powders has been developed [24,25]. Popular functional ingredients are considered—grains, pseudo-grains, legumes and vegetables, sources of dietary fiber, fish ingredients, herbs, inulin, resistant starch, and others—which are used to fortify pasta to obtain functional pasta with excellent technical quality and increased nutritional value [26,27].

Pumpkin is a natural means of cleansing the body, removing fluid, improving digestion, and functions as a diuretic. Pumpkin contains 4–5 times more β-carotene than carrots and, taking into account the high content of tocopherol (vitamin E) in pumpkin seeds and fruits, pumpkin can be called the most important product in restoring and maintaining sexual function in men [28,29]. The effect of adding pumpkin powder in different quantities (5, 7.5 and 10%) on the rheological properties of wheat dough, the quality of pasta preparation, as well as on sensory properties, was investigated. From the results obtained, it follows that the addition of pumpkin powder significantly increased the losses during cooking; the highest losses during cooking (6.6%) were noted after adding 10% pumpkin powder, and the addition of pumpkin powder increased the water absorption of pasta from 181.0% (control) to 211.2% (10% pumpkin powder) [30]. Increasing the proportion of pears, dates and apple by-products from 2.5 to 10% significantly changed the texture and structure of the pasta [31]. Adding oat bran to pasta increased the total dietary fiber content (16.43% w/g, g), while apple flour decreased the protein content (11.16% w/g, g). Pasta samples made with 50/50 durum wheat/oat-bran semolina and durum wheat/apple-flour semolina increased the antioxidant activity by ≈46% and ≈97%, respectively [32].

In recent years, ozone, ions, ion ozone, and electronic technology, which have several advantages over special additives and technologies, are increasingly used in the food industry. The use of ion-ozone technology agents that have many useful properties (bactericidal, redox, etc.) in food production is the latest trend and represents a promising direction in food production. Currently, the scientists of the Almaty Technological University are researching the use of ozonated, ionized, and ion-ozonated water in the production of flour, baked goods, pasta, confectionery products from wheat flour, and flour from a mixture of wheat, grains, oilseeds and legumes, which improves the quality and safety and environmental friendliness of finished products [33,34,35].

To reduce the deteriorating effect of corn and amaranth flour on the pasta properties of durum-wheat flour, ionized water was used. It has been established that the best quality of pasta is achieved when using ionized water with an ion concentration of 3000 units/cm^3^, ozone of 2 mg/L, with a dosage of amaranth flour of 17.5%, and 20% corn flour to pasta flour [36,37].

The advantages and detailed descriptions of ion-ozone treatment and ion-ozonated water are given in scientific works [38,39,40,41,42].

Thus, expanding the range of environmentally friendly products of increased nutritional and biological value is possible due to the additional introduction of processed grain products, fruits and vegetables, and ion-ozonated water into the pasta recipes, which will not only allow for the creation of a new generation of products aimed at preserving and improving public health, but will also involve additional raw materials in food circulation.

## 2. Materials and Methods

### 2.1. Objects of Study and Sample Preparation

To prepare pasta, whole-grain flour from Almaly soft wheat, pumpkin powder and ion-ozonated water were used in the work.

Whole-grain flour was obtained by grinding soft wheat of the Almaly variety in an SD-1 mill. The flour particle sizes were 300–400 microns. Then, it was treated with an ion-ozone flow in an ion-ozonator installation, while the concentration of ion ozone was 1.5–2.5 × 10^−6^ mg/cm^3^ (Table 1—X_1_).

Pumpkin powder is added to enrich, improve the biological value, and enhance the taste properties of pasta. Thanks to its valuable chemical composition, it enriches pasta with dietary fiber, minerals, organic acids, vitamins and natural dyes.

Pumpkin powder was obtained as follows: The pumpkin, which has dark orange flesh, was thoroughly washed and peeled, cut into pieces, and the seed nest was separated. The peeled and cut pumpkin was dried on a Hurakan HKN-DHD10 Dehydrator (Hurakan, Tallinn, Estonia) at a temperature of 110 °C for 7 h. Dried pumpkin pieces were ground in an SD-1 mill with a dispersion of 50–100 microns, while the moisture content of the powder was 9.5%. The total amount of waste and loss of dry matter when producing pumpkin powder is 25–30%. Pumpkin powder is a hygroscopic product with a pleasant sweetish taste and yellow-cream color.

Ion ozone with an ozone concentration of 2 mg/L and oxygen ions of 1000 units/cm^3^ was used to improve the consistency, dough properties, and gluten quality of finished products. Ion-ozonated water was obtained in an ion-ozonator installation.

To knead the dough, 600 g of whole-grain flour and pumpkin powder 1–3% were placed in the Gastrorag QF-3470 dough mixer (Table 1—X_3_); the unit was turned on, the required amount of ion-ozonated water was gradually added, and it was evenly distributed throughout the flour surface. The water temperature was 50–60 °C (Table 1—X_2_). With warm kneading, flour particles are moistened faster, gluten threads and films are formed and bound together, and the dough is more likely to form into lumps and is easily kneaded. The softness and plasticity of the dough, kneaded in warm water, make it easier and faster to form smoother products. After 5 min of kneading, stop the dough mixer to clean the spikes of the kneading blades from sticking dough. The total time required for complete mixing is 15–20 min. During this time, the dough turns into an elastic–plastic mass, seemingly consisting of individual slightly crumbling lumps. The consistency of the kneaded dough is determined by organoleptic indicators 5 min after the start of kneading. The moisture content of the dough fluctuated in the range of 29–33%.

When the dough is ready, open the valve and, using kneading blades, place the dough into the pressing chamber, where it is fed by a screw onto the matrix and pressed into pasta. The first curved piece of pasta, 5–7 cm long, was not suitable for further analysis, so it was cut off and discarded. The dried strands of pasta were placed on the table, cut into pieces 25 cm long, and placed in cassettes for drying.

On the day of making pasta, the temperature in the thermostat chamber was maintained at 36 °C and the relative air humidity was up to 80%; the holes on the top of the thermostat were opened. After the cassettes were fully loaded, a cuvette with water (1 L) at room temperature was placed in the thermostat chamber, the fan was turned on, one hole was closed, and the other was opened. Air entered only through the slots in the cap.

The next day, the hole at the top of the thermostat was closed, the fan was turned on, and the required drying temperatures were set to 50–60 °C (Table 1—X_4_). The active drying of the pasta was carried out continuously for 40 h, moist air was removed from the thermostat by periodically opening the door, and the relative humidity was 75%.

During this period, to achieve uniform drying, the cassettes containing pasta were turned 180 °C four times. On the third day of drying, in the morning, the water was poured out of the cuvette and the heating was turned off, but the fan continued to work. Within 7–8 h, the temperature in the thermostat gradually decreased to 25–27 °C, and accordingly, the relative air humidity dropped to 65–70%.

After drying is completed, the pasta was placed in a desiccator for resting. The final moisture content of the pasta should be no more than 13%.

### 2.2. Standard Research Methods

The organoleptic assessment of pasta products was carried out according to GOST 51865–2002 “Pasta products. General specifications” [43]. According to the following indicators: color, surface condition, shape, fracture, taste, smell, and the condition of the product after cooking.

The smell is determined before and after cooking. Take a pasta product, place it onto clean paper, warm it with your breath and examine it for smell.

To enhance the sensation of smell, the pasta product is transferred to a glass, hot water at a temperature of 60 °C is poured into the glass, then the water is drained and the smell is determined.

The smell of good-quality pasta products is weak, and pleasant. The smell of mold, mustiness and other foreign smells that arise from the improper storage of pasta and processing of poor-quality raw materials are to be avoided.

The taste of pasta should be slightly sweetish, without sour, bitter, or other foreign tastes. Foreign tastes in pasta are often caused by oxidation, rancidity, contamination by wormwood, or the presence of binders. An unusual taste and smell in products may arise as a result of their spoilage during storage, drying (dough souring), or when using poor-quality flour.

The color depends on the main and additional raw materials, the conditions of the technological process, and the nature of the grain. The color of pasta can be used to roughly judge its grade. The color of flour is determined by comparing the sample under study with the standard or with the description in the standard.

The color of the products is uniform, corresponding to the grade of flour. Products made from durum wheat have a yellow, white or slightly cream color—products made from bakery flour, or from flour of soft wheat (cream—for the extra grade, white—for the premium grade, white with a yellowish or grayish tint—for the first). The addition of tomato paste imparts an orange color, while spinach adds a greenish tint. Traces of under-mixing (white stripes and spots), as well as bran particles in the form of dark dots and spots should be avoided in the products.

The surface must be smooth, and the pasta must retain its intended shape. However, slight bends and curvatures of the products are allowed. The surface of premium-grade products must be smooth, while roughness is allowed for other grades. The fracture of the products must be glassy.

An important indicator is the condition of the pasta after cooking. To determine the condition of the pasta after cooking, 50 g of pasta is weighed and its volume is measured. For this purpose, 300 cm^3^ of tap water at room temperature is poured into a 500 cm^3^ graduated cylinder and the sample taken is immersed within.

The pasta must be completely covered with water. The cylinder is shaken to extract oxygen bubbles from it. The volume of the pasta (V_1_) is determined by the rise in the water level. Then, the water is drained, and the pasta is transferred to boiling water and cooked until done.

The maximum cooking time is as follows: for tubular products with a diameter of 5.5 mm—no more than 20 min; for tubular products with a diameter of 5.5 mm—no more than 25 min; for vermicelli with a diameter of 1.2 to 3 mm, as well as for noodles and shaped products—no more than 15 min; for vermicelli with a diameter of 1.2 mm—no more than 10 min.

After cooking, the pasta is transferred to a sieve, the water is allowed to drain, and the volume (V_2_) is measured again in the manner described above.

The increase in volume (C) is calculated using the formula:

(1) $$C=\frac{V_{2}}{V_{1}}$$

where:

C—the coefficient of increase in volume
V_1_—the volume before cooking
V_2_—the volume after cooking.

The condition of pasta after cooking, the formation of lumps, clumping, and loss of shape are determined; when cooking until done, the product should not lose shape, stick together, form lumps or fall apart at the seams. The water after cooking should not be cloudy, because this indicates a loss of nutrients. Important indicators of the quality of the products are their cooking ability and strength. Pasta after cooking for 10–20 min (depending on the type) until done should increase at least twofold in volume (in fact, they it increased by 3–4 times as much), be elastic, not sticky, and not form lumps. The cooking ability of products decreases somewhat with increasing shelf life [44].

A determination of the physicochemical indicators of pasta—humidity, acidity, ash content, shape retention, and amount of dry substances transferred into the cooking water—was carried out according to GOST 31964-2012 [45].

Humidity was determined by accelerated drying in an oven at a temperature of (130 ± 2) °C for 40 min.

The mass fraction of moisture W, %, is calculated using the following formula:

(2) $$W=\frac{m_{1}-m_{2}}{m}\times 100$$

where:

m_1_—the mass of the bottle with the sample for analysis before drying, g;
m_2_—the mass of the bottle with the sample for analysis after drying, g;
m—the mass of the sample for analysis, g.

Acidity was determined by titrating an aqueous suspension of ground pasta with sodium hydroxide.

Acidity X, in degrees, is calculated using the following formula:

(3) $$X=\frac{V\times 20}{10}\times K$$

where:

V—the volume of sodium hydroxide solution consumed for titration of 100 g of pasta, cm^3^;
20—the conversion factor per 100 g of pasta;
10—the conversion factor 0.1 n. 1 N sodium hydroxide solution;
K—the correction factor to titer 0.1 n. sodium hydroxide solution.

Ash content was determined using a 10% HCI solution. The procedural essence of this method is to treat the ash with a 10% solution of hydrochloric acid while heating, filtering the solution and burning the sediment on the filter in a muffle furnace.

The mass fraction of ash insoluble in a 10% solution of hydrochloric acid per dry weight, %, is calculated using the following formula:

(4) $$X_{1}=\frac{\left(m_{1}-m_{2}\right)}{m}\times \frac{100}{\left(100-W\right)}\times 100$$

where:

m_1_—the mass of the crucible with the residue on the filter after calcination, g;
m_2_—the mass of an empty crucible with filter ash, g;
m—the mass of the sample for analysis, g;
W—the mass fraction of moisture in the test sample for analysis, %.

Form retention was determined in the following order: Pour 1000 cm^3^ of distilled water into a cooking vessel and bring to a boil. A sample for analysis, taken from a laboratory sample of pasta in the amount of 50 g (based on the whole product), is immersed in boiling water and cooked, stirring until the water boils again. The cooked pasta is transferred to a sieve, the cooking water is allowed to drain, and the pasta is laid out on a plate. Through the external inspection of cooked pasta, the number of products that have not retained their original shape is determined.

The shape retention of pasta X_2_, %, calculated by the following formula:

(5) $$X_{2}=\frac{A}{B}\times 100$$

where:

A—the number of pasta pieces that retained their shape after cooking, pcs;
B—the number of pasta pieces selected for cooking, pcs.

The amount of dry matter that passed into the cooking water was determined by evaporation in a water bath and accelerated drying in an oven at a temperature of 130 °C for 30 min.

The mass of dry matter transferred during the cooking of pasta into cooking water X_3_, %, is calculated using the following formula:

(6) $$X_{3}=\frac{(A-B)\times V_{1}}{V_{2}\times a}\times \frac{100}{100-W}\times 100$$

where:

A—the mass of the evaporation cup with dry residue, g;
B—the mass of an empty cup for evaporation, g;
V_1_—the total volume of cooking water of the test solution, cm^3^;
V_2_—the volume of cooking water of the test solution taken for evaporation, cm^3^;
a—the mass of the sample for analysis, g;
W—the humidity of the test sample for analysis, %.

The coefficient of increase in the mass of products (C_m_), the coefficient of increase in the volume of products (C_v_), and the duration of cooking until done are determined in accordance with the manual [46].

The amount of absorbed water is characterized by the coefficient of increase in mass (volume) of pasta C during cooking. This coefficient is determined by the following formula:

(7) $$C=\frac{m_{2}}{m}$$

where:

m_2_—the mass (volume) of welded products, g (determined after draining the cooking liquid);
m—the mass (volume) of dry products, g.

The duration of cooking until done is determined by the period of time from placing the products in boiling water to the disappearance of the powdery, uncooked layer. To check for doneness, during cooking, periodically remove a piece of pasta from the pan, place it between two glass slides and squeeze gently.

Protein content was determined according to GOST 10846-91 [47]. The essence of the method is the mineralization of organic matter with sulfuric acid in the presence of a catalyst with the formation of ammonium sulfate, the destruction of ammonium sulfate with alkali with the release of ammonia, and the distillation of ammonia with water vapor into a solution of sulfuric or boric acid, followed by titration.

When distilling ammonia into a sulfuric acid solution, the nitrogen content (N_1_) at actual humidity in percent is calculated using the following formula:

(8) $$X_{2}=\frac{(V_{0}-V_{1})\times K\times 0.0014\times 100}{m}$$

where:

m—the mass of the sample, g;
V_0_—the volume of 0.1 mol/dm^3^ sodium hydroxide solution used for titration of 0.05 mol/dm^3^ sulfuric acid in a “blank” determination, cm^3^;
V_1_—the volume of 0.1 mol/dm^3^ sodium hydroxide solution used for titration of 0.05 mol/dm^3^ sulfuric acid in the analyzed solution, cm^3^;
K—the correction to the titer of 0.1 mol/dm^3^ sodium hydroxide solution:
0.0014—the amount of nitrogen equivalent to 1 cm^3^ of 0.05 mol/dm^3^ sulfuric acid solution, g.

The mass fraction of fat is determined according to GOST 29033-91 [48]. The essence of the method is to extract crude fat from the product with a solvent, then remove the solvent, and dry and weigh the extracted fat.

The mass fraction of fat in each sample of the product as a percentage in terms of dry matter is calculated using the following formula:

(9) $$X=\frac{(m_{2}-m_{1})\times 100\times 100}{m\times (100-W)}$$

where:

m—the mass of the product sample, g;
m_1_—the mass of the empty flask, g;
m_2_—the mass of the flask with fat, g;
W—the product humidity, %.

The starch content is determined according to GOST 10845-98 [49]. The essence of the polarimetric method for determining starch is the dissolution of starch contained in the grain or its processed products in a hot diluted solution of hydrochloric acid, the precipitation and filtration of the dissolved protein substances, and then measuring the optical angle of rotation of a starch solution.

The starch content in the grain or its processed products of (X) in terms of dry matter as a percentage is calculated using the following formulas:

− when using a polarimeter with a dial scale:

(10) $$X=\frac{aK100}{0.3468(100-W)}$$

− when using a saccharimeter with a normal scale:

(11) $$X=\frac{aK100}{100-W}$$

where:

a—the reading of a polarimeter or saccharimeter, scale degree;
W—the moisture content of grain or its processed products, %;
K—the conversion factor for grain and its processed products is, respectively, equal to wheat—1.898.

The crude fiber content was determined according to GOST 31675-2012 [50]. The method is based on the sequential processing of a weighed test sample with solutions of acid and alkali, followed by ashing and the gravimetric determination of the organic residue. The crude fiber content is expressed as a mass fraction in percent or grams per 1 kg of dry matter.

The vitamin A content was determined according to GOST R 54635-2011 [51]. This standard establishes a method for determining the mass fraction of vitamin A in the form of retinol, retinol acetate, and retinol palmitate using high-performance liquid chromatography (HPLC). The measurement range for the mass fraction of vitamin A is from 0.5 to 10.0 ppm. The determination of vitamin A in the extract obtained from the analyzed sample is carried out by HPLC separation followed by photometric or fluorometric detection. If necessary, the extract is obtained after alkaline hydrolysis of the analyzed sample. Quantitative analysis is carried out by the external standard method using the area or height of the peaks of retinol, retinol acetate, and retinol palmitate.

The content of vitamin C is determined according to GOST 34151-2017 [52]; this standard establishes a method for determining vitamin C in food products using high-performance liquid chromatography (HPLC). The content of vitamin C is determined as the sum of the L(+)-ascorbic and L(+)-dehydroascorbic acids. The method is based on the extraction of vitamin C from a sample with a solution of metaphosphoric acid, followed by the reduction in L(+)-dehydroascorbic acid to L(+)-ascorbic acid and the determination of the total content of L(+)-ascorbic acid using high-performance liquid chromatography (HPLC) with spectrophotometric detection at a wavelength of 265 nm.

The vitamin E content was determined according to GOST EN 12822-2014 [53]. This standard specifies a method for the determination of vitamin E in foods by high-performance liquid chromatography (HPLC). The method is based on the determination of α-, β-, γ- and δ-tocopherols in a sample solution by HPLC with photometric (ultraviolet) or, preferably, fluorometric detection. To prepare a sample solution, in most cases it is necessary to saponify the sample material, followed by an extraction of the analytes. The compounds being determined are identified by retention time values and quantified using an external standard based on the results of measurements of peak areas or peak heights.

The β-carotene content was determined according to GOST EN 12823-2-2014 [54]; this standard establishes a method for the determination of total β-carotene in food products using HPLC. The amount of β-carotene isomers in the test sample solution is determined by HPLC using a spectrophotometric detector in the visible range. The extract obtained after saponification as described in GOST EN 12823-1-2020 [55] can be used for quantitative analysis.

The content of mineral substances of iron, calcium, potassium, magnesium, and zinc was determined according to GOST 32343-2013 [56]. This standard establishes a method for the determination of calcium, copper, iron, magnesium, manganese, potassium, sodium and zinc using atomic absorption spectrometry. The essence of the method is to dissolve the analyzed sample in hydrochloric acid, if necessary with ashing in a muffle furnace at a temperature of (550 ± 15) °C, remove the present silicon compounds by precipitation and filtration, and then atomize the resulting solution in an acetylene flame. The absorption of each element in the analyzed solution is measured in comparison with the absorption of the same element in the calibration solution.

### 2.3. Methodology for Processing the Results of Experimental Studies

This study aims to investigate the influence of the following 4 factors on the quality of pasta: ion-ozone concentration in ion-ozonated water, water temperature for kneading pasta dough, pumpkin powder content, and pasta drying temperature. To reduce the number of experiments and obtain a reliable assessment of the influence of individual factors on the quality indicators of pasta, multifactorial experimental design methods were used.

Data processing and all necessary calculations were carried out using the PLAN sequential regression analysis program developed at the Odessa National Academy of Food Technologies [57,58]. This program enables the calculation of regression coefficients for each quality indicator, testing their significance, and—after eliminating all insignificant coefficients—deriving the necessary statistical characteristics of the resulting regression equations, including checking their adequacy of experimental data.

The calculations of regression coefficients were carried out using matrices in natural dimensions and, accordingly, the equations were also obtained in natural dimensions.

The general form of the equations for the obtained regression equations is as follows:

− for 4 factors:

y = b_0_ + b_1_C + b_2_P + b_3_w + b_4_τ + b_12_C × P + b_13_C × w + b_14_C × τ + b_23_P × w + b_24_P × τ + b_34_w × τ (12)

The total number of samples studied was 4^2^ = 16 pieces. The designations of the variables in these equations are as follows:

y—quality indicators of pasta;
C—ratio of ion concentration (units/cm^3^) to ozone concentration (g/cm^3^), units/g;
P—excess pressure (cavitation), ati;
w—humidity before pasta processing, %;
τ—processing time, min.

To numerically estimate the coefficients in the regression equations in the PLAN program, the least squares method (LSM), implemented in matrix form, was used.

Since the experiments were carried out once, duplicate (parallel) experiments were carried out in the center of each studied sample to assess the dispersion of the reproducibility of the results, and based on the results, the dispersion of the reproducibility and, accordingly, the root mean square errors of the experiments sy were determined for each of the 16 experiments.

The significance of the b_i_ coefficients was tested using a confidence interval, which was calculated using the formula:

(13) $$\varepsilon_{b_{i}}=t_{cr}\sqrt{S_{b_{i}}^{2}}$$

where: $S_{b_{i}}^{2}$ —variance of the *i*-th regression coefficient.

If the condition was met $|bi|\geq \varepsilon_{b_{i}}$, then the coefficient was considered statistically significant for the accepted significance level (0.05).

Since the regression coefficients were calculated using matrices in natural dimensions, after removing the most insignificant coefficient, the remaining significant coefficients and their confidence intervals were recalculated. This procedure, in accordance with the principle of sequential regression analysis, was carried out until there was not a single insignificant coefficient left in the regression equation.

The resulting regression equation was assessed by the relative errors between the empirical and calculated values as follows:

(14) $$\delta =\frac{\left|\bar{y}-\hat{y}\right|}{\bar{y}}$$

However, more accurate conclusions about the suitability of the resulting equation for practical use can be made after its statistical analysis, based on estimates of variances. For this purpose, the resulting equation, from which all insignificant coefficients were excluded, was checked for adequacy to the experimental data.

The adequacy test was carried out using the Fisher criterion, which is the ratio of the larger variance to the smaller one, i.e.,

(15) $$F=\frac{S_{\mathrm{inad}}^{2}}{S_{\bar{y}}^{2}}\;(\mathrm{at}\;S_{\mathrm{inad}}^{2}>S_{\bar{y}}^{2})\;\mathrm{or}\;F=\frac{S_{\bar{y}}^{2}}{S_{\mathrm{inad}}^{2}}\;(\mathrm{at}\;S_{\bar{y}}^{2}>S_{\mathrm{inad}}^{2})$$

where

$S_{\mathrm{inad}}^{2}$—the dispersion of inadequacy, which characterizes the “scatter” of values calculated using the equation $\bar{y_{u}}$ and the results of the experiments (measurements) $\bar{y_{u}}$;
$S_{\bar{y}}^{2}$—the dispersion of the experimental error, which characterizes the “scatter” of the values of yu in parallel experiments and the average value of the experiments $\bar{y_{u}}$.

The calculated value of the Fisher criterion obtained from one of the above expressions was compared with the tabulated (critical) value of Fcr. In the case when F < Fcr, the resulting equation adequately describes the experimental data with an accepted reliability of 95%.

All regression coefficients for the studied pasta samples, as well as their statistical characteristics, were obtained as a result of the PLAN program. The designations in the PLAN program listings are adopted as follows:

b—regression coefficients;
e—confidence intervals for the corresponding coefficients;
x_1_, x_2_, x_3_, x_4_—designations of the first, second, third, etc., factors;
Yex—experimental values of quality indicators “U”;
Yc—calculated (according to the obtained regression equation, from which all insignificant coefficients have been removed) values of the quality indicator “Y”;
styu—relative deviation (in%) of the calculated Yp and experimental Ycp values of the quality indicator “U”;
tcr—the critical (tabular) value of Student's test (for a significance level of 0.05);
s2y, s2ag—weighted average dispersion of the average experimental results s2y and dispersion of inadequacy s2ad, respectively;
sy, sag—the same, but standard deviations;
Ns2y, Ns2ag—the number of degrees of freedom;
Fc, Fcr—calculated and critical (tabular) values of the Fisher criterion.

The adequacy of the equations to the experimental data was checked at a significance level of 0.05.

## 3. Results and Discussion

To study the effect of ion-ozonated water on the properties of the dough and the finished product made from whole-grain flour from Almaly soft wheat and pumpkin powder, 16 samples of pasta were prepared, and the organoleptic and physicochemical indicators, as well as the structural, mechanical, and technological properties, were studied. As a control sample, pasta was prepared made from whole-grain flour from soft wheat of the Almaly variety without the use of pumpkin powder and ion-ozonated water.

The chemical composition of pumpkin powder and whole-grain soft wheat flour of the Almaly variety are given in Table 1.

A study of pumpkin powder and whole-grain soft wheat flour of the Almaly variety showed that they contain a fairly large amount of proteins, carbohydrates, vitamins, Beta carotene and minerals. It was found that flour from soft wheat of the Almaly variety does not contain vitamins A and C.

**Figure 1:**
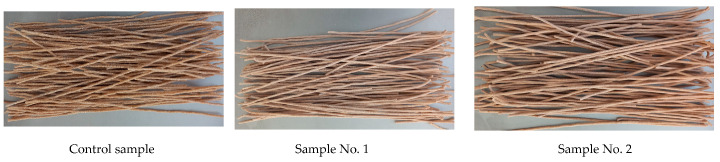

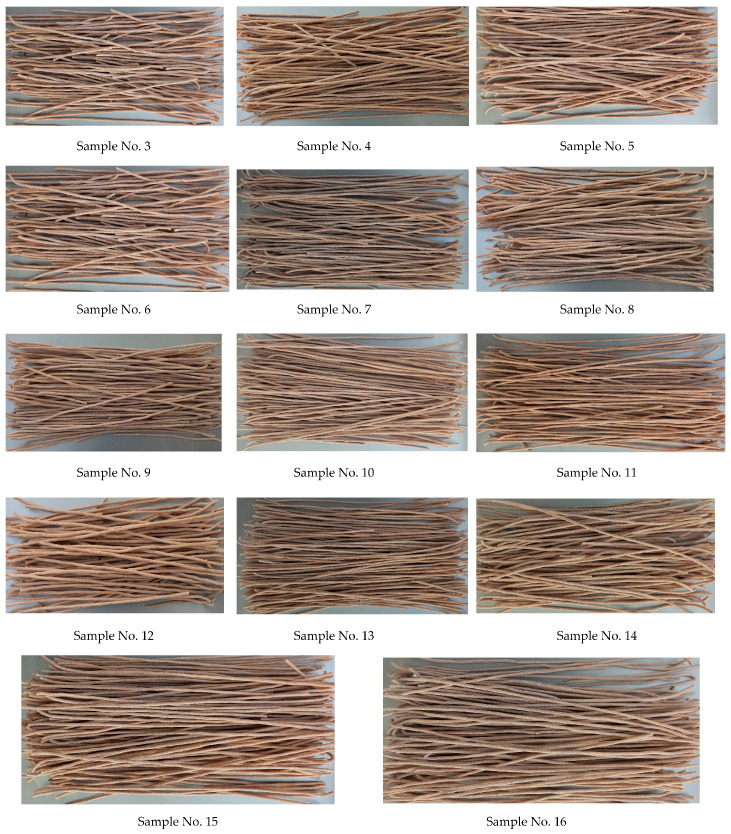
Control sample and test samples of pasta made from whole-grain flour from Almaly soft wheat and pumpkin powder using ion-ozonated water.

The external appearance of pasta is shown in Figure 1.

The organoleptic parameters were also studied, and a visual and tasting assessment was carried out using a 100-point scale, with five experts participating; the results are shown in Table 2.

From the data in Table 2 and Figure 1, it is clear that the surface conditions of the control sample and sample No. 4 are slightly rough (80%), while samples No. 2 and No. 14 are smooth (70%), but exhibit minor cracks. The remaining samples had smooth surfaces (100%). The shape of all samples was correct (100%).

The colors of the pasta were different: the control sample and samples No. 5—No. 8 were cream-colored (80%), samples No. 13—No. 16 were lightly cream-colored (90%), samples No. 1–No. 4 were cream-colored with a yellowish tint (95%), samples No. 9–No. 12 were lightly cream-colored with a yellowish tint (100%). The taste of the products when using 3% pumpkin, respectively, produced a barely noticeable pumpkin taste. The smell of all samples was characteristic.

Having analyzed the data in Table 2, we can conclude that, in accordance with GOST 51865–2002 “Pasta products. General specifications”, samples No. 9-No. 12 showed the best results, as all organoleptic indicators—surface condition, shape, color, taste, and smell—were 100%.

In the conducted studies, we studied the dependence of the quality indicators of pasta made from whole-grain flour from Almaly soft wheat using fine additives from plant raw materials and ion-ozonated water on the following technological regimes for the production of pasta:

x_1_—concentration of ion ozone in ion-ozonized water C_io_ × 10^−6^, mg/cm^3^;
x_2_—water temperature for kneading pasta dough t_w_, °C;
x_3_—content of pumpkin powder C_pp_, %;
x_4_—pasta drying temperature t_d_, °C.

To reduce the number of experiments and obtain reliable results from experimental studies, the methods of planning multifactorial experiments were used.

The influence of several factors on the quality indicators of pasta products made from whole-grain flour from Almaly soft wheat and pumpkin powder using ion-ozonated water was studied, listed as follows: ion-ozone concentration, water temperature, pumpkin powder content, and drying temperature. As a result of the four-factor experiment, 16 experimental samples were obtained. Data on the factors are given in Table 3.

The following quality indicators were determined in the studied samples of pasta: chemical composition (y_1_–y_8_), rheological properties (y_9_–y_11_), cooking properties (y_12_–y_16_), vitamin, Beta carotene and mineral composition (y_17_–y_25_):

y_1_—the moisture content of pasta, %;
y_2_—the acidity of pasta, degrees;
y_3_—the protein content, %;
y_4_—the starch content, %;
y_5_—the carbohydrate content, %;
y_6_—the fat content, %;
y_7_—the fiber content, %;
y_8_—the ash content, %;
y_9_—the total deformation H_1_, mm;
y_10_—the plastic deformation H_2_, mm;
y_11_—the elastic deformation H_3_, mm;
y_12_—the preservation of the shape of pasta, %;
y_13_—the coefficient of increase in mass of products (C_m_);
y_14_—the coefficient of increase in the volume of products (C_v_);
y_15_—the amount of dry matter transferred into cooking water, %;
y_16_—the duration of cooking until done, min;
y_17_—the vitamin A content, mg/100 g;
y_18_—the vitamin E content, mg/100 g;
y_19_—the vitamin C content, mg/100 g;
y_20_—the content of Beta carotene, mg/100 g;
y_21_—the calcium content, mg/100 g;
y_22_—the potassium content, mg/100 g;
y_23_—the magnesium content, mg/100 g;
y_24_—the iron content, mg/100 g;
y_25_—the zinc content, mg/100 g.

To reduce the influence of uncontrolled factors on the results of the experiments, the experiments were randomized using tables of random numbers.

The physical and chemical parameters of the finished pasta products were determined before and after cooking, namely humidity and acidity, with the cooking properties listed as follows: the preservation of the shape of the cooked products, the coefficient of increase in the mass of products (Cm), the coefficient of increase in the volume of products (C_v_), the amount of dry substances transferred into the cooking water, and cooking time until done.

The moisture content of pasta is an important indicator of commercial quality, which determines the survivability of products in long-term storage without spoilage. It is also the main factor determining the yield of finished products, that is, the consumption of flour to produce 1 ton of products. The moisture content of pasta in all samples was no higher than 13%.

Acidity is a quality indicator that characterizes the taste properties and degree of freshness of pasta products. Acidity is determined primarily by the acidity of the original flour. The acidity of pasta made from whole-grain flour from durum and soft wheat, as well as the finely dispersed additives from vegetable raw materials and ion-ozonated water, was in the range of 3.4–4.0 degrees, which satisfies the requirements of the standard.

The results of the chemical properties of pasta products made from whole-grain durum-wheat flour using pumpkin powder and ion-ozonated water are presented in Table 4.

Table 3 shows that the moisture content of pasta ranged from 12.4 to 13.0%. The acidity fluctuated from 3.4 to 4.0 degrees, with the lowest levels shown by samples No. 4, No. 9, and No. 13. The best indicator of the quality of pasta is the highest protein content with a minimum amount of dry matter transferred into the cooking water. Maximum protein retention was observed in experiments No. 6, No. 7, and No. 11, and minimal losses of dry matter were observed in experiments No. 10, No. 11, and No. 12. However, in experiment No. 11, the best results were achieved—a maximum protein content of 14.45% and a minimum loss of dry matter of 5.9%. Starch content varied from 65.30 to 69.76%, with the highest amounts being shown by samples No. 5–8, No. 13, and No. 16. Carbohydrate content varied from 70.08 to 75.94%, with the highest amounts being observed in samples No. 5–8, No. 11, No. 13–16. Fat content varied from 2.51 to 3.52%, with the lowest amounts being shown by samples No. 5–8, No. 11, and No. 13–16. Fiber content varied from 1.18 to 3.78%, with the highest amounts being observed in samples No. 1–4, No. 10, No. 12, and No. 14. Ash content ranged from 2.07 to 4.78%, with the highest amounts found in samples No. 1–No. 4, No. 9, No. 10, and No. 12.

At present, the deformation of physical bodies and colloidal, dispersed systems, similar to flour dough, is controlled by rheological methods. To produce high-quality products, pasta dough must be sufficiently strong and have optimal elastic–plastic properties. All these properties are determined mainly by three main factors: the quality of flour, the parameters of kneading the dough, and its pressing. The quality of pasta products ultimately depends on the rheological properties of pasta dough. The rheological properties of semi-finished products during the technological process are continuously changing under the influence of a set of processes leading to a change in the state and structure of the components of the raw materials, the ratio of the liquid and solid phases of semi-finished products, and the activity of dough enzymes. The processing of products is accompanied by complex physicochemical and mechanical processes. Flour dough is a complex heterogeneous colloidal dispersed system. Adding various additives to the dough improvers can change its rheological properties, relaxing and improving the consistency of the dough. To study the effect of dosages of finely dispersed additives from plant raw materials and ion-ozonated water on the rheological properties of dough made from whole-milled flour from hard and soft wheat, the change in the physical properties of the dough was assessed using a structurometer. The rheological properties (total, plastic, and elastic deformations) of the dough during the preparation of pasta samples given in Table 5 were determined using a structurometer.

The results obtained for the elastic and plastic deformation of pasta made from whole-grain flour and pumpkin powder indicate the significant role of ion-ozonated water in the adsorption binding of moisture, strengthening the structural and mechanical properties of the dough and finished products.

The study of the plastic and elastic deformation of dough made from whole-grain flour from durum and soft wheat, as well as finely dispersed additives from vegetable raw materials and ion-ozonated water, showed the same pattern as when studying the quality of finished pasta, that is, the values of the physical properties of the samples No. 9, 10, 11, 12 are better than in other samples. The test samples numbered 2, 4, and 14 were characterized by low performance.

The cooking properties of pasta were also investigated, and the results are shown in Table 6.

The duration of cooking until done in pasta made from whole-grain soft wheat flour was from 12 to 15 min, and from durum wheat—from 13 to 16 min. The coefficient of increase in the mass of pasta varied accordingly, from 2.12 to 2.51 and from 1.78 to 2.23. Closely related to these indicators is the main indicator of the cooking properties of pasta—the amount of dry substances transferred into the cooking water. Thus, the value of this indicator varied, respectively, from 5.9 to 7.0 and from 5.76 to 6.41%.

Next, the vitamin and mineral compositions of finished pasta products were studied; the results are shown in Table 7.

Assessing the results of the study of the elemental composition of pasta, we can conclude that pasta made from whole-grain soft wheat flour using pumpkin powder is complete in comparison with control samples in terms of the content of magnesium, iron, calcium, potassium and zinc. Thus, the iron content increased in samples of pasta made from whole-grain flour from Almaly soft wheat with the addition of pumpkin powder from 1.9 to 3.12 mg, while the control sample was 1.15 mg. The calcium content increased from 117.97 to 197.53 mg, the control is 100.51 mg, etc.

In pasta, there was an increase in their vitamin value. Thus, according to the analysis of the presented data, the use of even small quantities of pumpkin powder is advisable for enriching pasta with valuable nutritional components.

Thus, a study of the quality of pasta made from whole-grain flour from durum and soft wheat, as well as finely dispersed additives from plant raw materials and ion-ozonated water, showed that the values of the quality indicators of the finished products of samples No. 9, No. 10, No. 11, and No. 12 are better than in other samples. Samples No. 2, No. 4, and No. 14 were characterized by low-quality indicators.

Based on the experimental data obtained while studying the influence of finely dispersed additives from plant raw materials and ion-ozonated water on the rheological properties of the dough and the quality of finished products made from whole-grain soft wheat flour, the optimal dosages of the additive (pumpkin powder) and technological modes for preparing dough and drying raw pasta were established. 

Based on the results of the research, regression equations were obtained that adequately (according to the Fisher criterion) describe the influence of technological modes for the production of pasta C_io_, t_w_, C_pp_, and t_d_ on the above indicators of their quality. The optimal values of the influencing factors for this experiment are as follows (sample obtained by mode No. 11): x_1_ = 2.5 × 10^−6^ mg/cm^3^, x_2_ = 50 °C, x_3_ = 3.0%, x_4_ = 50 °C.

To determine the variance in experimental errors (reproducibility), in the center of the experimental plan, three parallel experiments were carried out, which are mentioned in the research methodology.

The calculations of regression coefficients were carried out using matrices in natural dimensions and, accordingly, the equations themselves were also obtained in natural dimensions.

The general form of regression equations for four factors is as follows:

y_i_ = b_0_ + b_1_C_o_ + b_2_t_w_ + b_3_C_pp_ + b_4_t_d_ + b_12_C_o_t_w_ + b_13_C_o_C_pp_ + b_14_C_o_t_d_ + b_23_t_w_C_pp_ + b_24_t_w_t_d_ + b_34_C_pp_t_d_, (16)

where:

y_i_—the *i*-th indicators of the quality of dough and the bread made from it;
C_io_—the concentration of ion ozone in ion-ozonated water, mg/cm^3^;
t_w_—the water temperature for kneading pasta dough, °C;
C_pp_—the content of pumpkin powder, %;
t_d_—the pasta drying temperature, °C.

Summary data on the obtained regression equations in natural variables are given in Table 8, Table 9, Table 10 and Table 11. The same table shows the mean square errors of the experiments Se and the inadequacy of S_inad_., as well as the calculated Fc and critical Fcr values of the Fisher criterion, indicating that all the obtained equations adequately describe the experimental data at confidence level *p* = 0.05.

The compiled regression equations are mathematical models that enable the prediction of the quality indicators of the processed dough and the products obtained from it, depending on the values of the technological modes of dough processing, i.e., the factors C_io_, t_w_, C_pp_, and t_d_.

From the data in Table 8, Table 9, Table 10 and Table 11, it is evident that, of the 25 studied indicators of the quality of pasta, only their acidity y_2_ and the coefficient of increase in the mass of products y_13_ do not depend on their production modes. Several indicators of pasta quality depend on one operating factor. The content of pumpkin powder C_pp_ determines the starch content y_4_, carbohydrate content y_5_, fat content y_6_, as well as the content of minerals—calcium y_21_ and magnesium y_23_. The drying temperature t_d_ of pasta depends on its moisture content y_1_.

Only the iron content y_24_ depends on two regime factors of the concentration of ion ozone in ion-ozonized water C_io_ and the content of pumpkin powder C_pp_, and such quality indicators as the preservation of the shape of pasta y_12_ and the magnification factor depend on the content of pumpkin powder C_pp_, the drying temperature of pasta t_d_, the volume of products y_14_, the duration of cooking until ready y_16_, and protein content y_3_.

The amount of dry substances transferred into the cooking water y_15_ and the ash content y_8_ depend on three regime factors, such as the concentration of ion ozone in ion-ozonated water C_io_, the content of pumpkin powder C_pp_, and the drying temperature of pasta t_d_. Only the potassium content y_22_ depends on the temperature of the water for kneading the pasta dough t_w_, the content of pumpkin powder C_pp_, and the drying temperature of pasta t_d_.

Finally, a large number of quality indicators depend on all four considered factors: three types of deformation of pasta (full y_9_, plastic y_10_ and elastic y_11_), fiber content y_15_, the content of the studied vitamins A, E, C and Beta carotene (y_17_, y_18_, y_19_ and y_20_, respectively), as well as the content of such minerals as zinc y_25_.

To optimize technological regimes for the production of pasta, the following indicators of their quality were chosen as target functions:

− the protein content (%):
  y_3_ = 5.551 + 3.481 × C_pp_ + 0.1569 × t_d_ − 0.06938 × C_pp_ × t_d_ → max; (17)
− the amount of dry substances transferred into cooking water (%):
  y_15_ = 6.112 − 0.177 × C_io_ × 10^−6^ × C_pp_ + 0.00742 × C_pp_ × t_d_ → min. (18)

An analysis of Equation (18) shows that the protein content is influenced by only two regime factors—the content of pumpkin powder C_pp_ and the water temperature for kneading pasta dough t_w_—and this influence manifests itself in the form of a paired contradictory mutual influence of these factors (the paired interaction coefficient is −0.06938 × C_pp_ × t_w_). Because of this, using the above equation in natural variables makes it difficult to analyze the influence of each factor C_pp_ and t_w_ on protein content.

**Figure 2:**
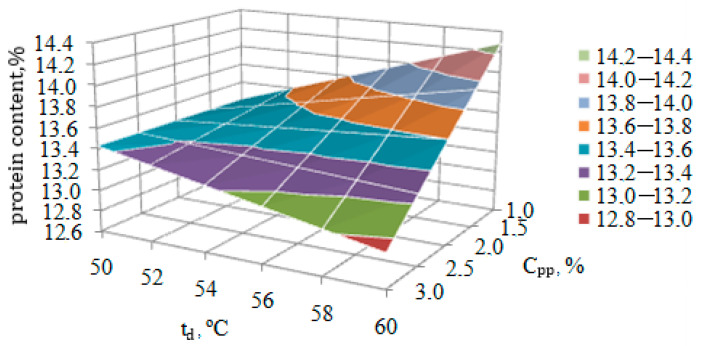
The response surface of the joint influence of factors C_pp_ and t_d_ on the protein content in pasta.

The clear nature of the joint mutual influence of factors C_pp_ and t_d_ on protein content (y_3_) can be determined from the response surface (Figure 2), constructed according to the above Equation (17).

Figure 2 shows the contradictory influence on the protein content in pasta of the content of pumpkin powder C_pp_ and their drying temperature t_d_. Thus, when pumpkin powder is introduced into pasta, C_pp_ ranges from 1.0% to 3.0% and the temperature t_d_ = 50 °C is used for drying; the protein content remains relatively unchanged, at 13.4%.

However, when using a temperature of 60 °C for drying pasta, a significant influence of the content of pumpkin powder C_pp_ added to the pasta dough on the protein content in pasta appears. So, if the amount of protein when introducing C_pp_ = 1% increases to 14.3%, then when pumpkin powder is introduced in the amount of C_pp_ = 3%, the protein content decreases by 35.3% and amounts to only 12.9%.

As for the influence of the drying temperature of pasta t_d_, its effect on the protein content in pasta also significantly depends on the input of pumpkin powder C_pp_. When entering C_pp_ = 1%, an increase in temperature from 50 to 60 °C increases the amount of protein by 0.87%, and when entering 3.0%, on the contrary, it decreases by 1.35%.

Thus, to increase the protein content in pasta products, it is necessary to reduce the input of pumpkin powder C_pp_ into their composition and increase their drying temperature t_d_. The remaining two factors C_io_ and t_d_ do not affect the protein content in pasta.

**Figure 3:**
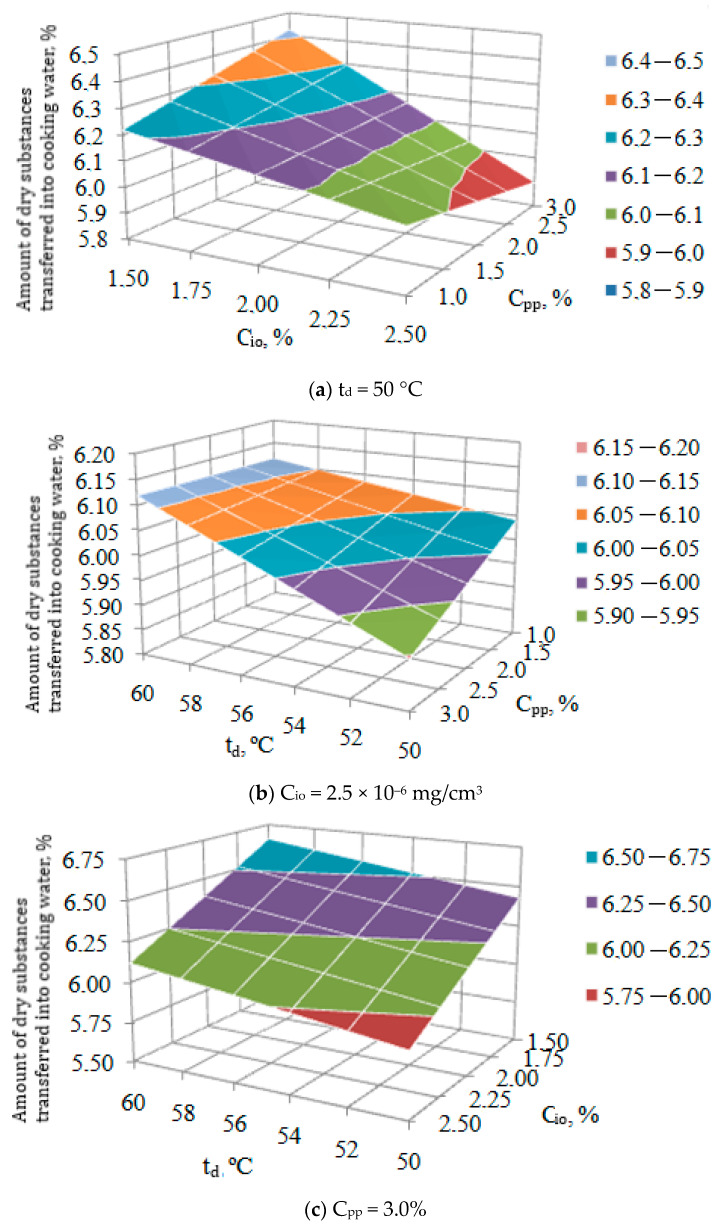
The response surface of the joint influence of factors C_io_, C_pp_, t_d_ on the content of dry substances in pasta.

The second objective function is the amount of dry matter transferred into the cooking water, which depends on the factors C_io_, C_pp_, and t_d_ and is shown in Figure 3.

Analysis of the given response surfaces shows the following.

Regardless of the content of introduced pumpkin powder C_pp_, an increase in the concentration of ion ozone C_io_ (Figure 3a) leads to a decrease in the amount of dry matter transferred into the cooking water. Furthermore, it is observed that the transition (loss) of dry substances into cooking water at C_io_ = 2.5 × 10^−6^ mg/cm^3^ will decrease with an increase in the introduced pumpkin powder. It is also worth noting that at low concentrations of ion ozone (C_io_ = 2.5 × 10^−6^ mg/cm^3^), an increase in the content of pumpkin powder leads, on the contrary, to an increase in the transfer of dry substances into cooking water.

The same nature of the influence of C_pp_ on the loss of dry substances is visible in Figure 3b at a drying temperature of 50 °C. Although less intense, this trend persists when C_pp_ decreases to 1%. Thus, lowering the drying temperature makes it possible to reduce the amount of dry substances passing into the cooking water.

Figure 3c shows the effect of the concentration of ion ozone C_io_ and drying temperature on the loss of dry substances. Since there is no significant effect of their interaction between these factors, the response surface has a linear character, which is visible in Figure 3c. This figure confirms the need to reduce the drying temperature t_d_ and increase the concentration of the ion ozone C_io_, which makes it possible to reduce the amount of dry substances passing into the cooking water (y_6_).

Thus, to reduce the loss of dry substances passing into the cooking water, it is necessary to increase the concentration of ion ozone C_io_, as well as reduce the content of pumpkin powder introduced into the pasta C_pp_ and its drying temperature t_d_.

The optimization of the pasta production process was carried out, taking into account the restrictions on the quality indicators of finished pasta.

The range of values of the regime factors C_io_, t_w_, C_pp_, and t_d_ was measured to ensure they correspond to the experiment planning matrix. The nonlinear programming method was used for optimization. The given restrictions on the quality indicators of pasta from y_1_ to y_25_ are consistent with the technical specifications and standards provided for in pasta production technology.

The calculations have shown that the maximum amount of protein in finished pasta is provided by the following optimal technological regimes:

− pumpkin powder content C_pp_ = 1.0%;
− pasta drying temperature t_d_ = 60 °C.

Under these optimal conditions, the calculated value of the objective function (protein content) is 14.28%. The concentration of ion ozone C_io_ and the temperature of the water for kneading pasta dough do not affect the protein content.

The minimum amount of DM transferred into the cooking water is ensured by the following optimal technological regimes:

− concentration of ion ozone in ion-ozonized water C_io_ = 2.5 × 10^−6^, mg/cm^3^;
− pumpkin powder content C_pp_ = 3.0%;
− pasta drying temperature t_d_ = 50 °C.

Under these optimal conditions, the calculated value of the objective function (loss of DM transferred into cooking water) is 5.90%. The water temperature for kneading pasta dough, t_w_, does not affect the protein content.

The values of other quality indicators of pasta under optimal conditions for the amount of protein y_3_ and the amount of dry matter transferred into the cooking water y_15_ are shown in Table 12.

The data in Table 11 indicate that the simultaneous optimization of the pasta production process using two optimally selected functions y_3_ and y_15_ results in different optimal modes. In this case, one of the two optimality functions under consideration (y_3_ or y_15_) must be taken as the main one, and the second must be used as a constraint on the corresponding quality indicator.

An analysis of literary sources indicates that the use of whole-grain flour and pumpkin powder, containing protein, fiber, vitamins, and mineral substances necessary for the human body, will solve the problem of meeting the needs of the population for high-quality food products with high nutritional and biological value, and also constitute one of the potentially protective components. Refs. [59,60] have established that whole-grain products have a significant protective effect on the body and lead to a reduction in the risk of cancer, weight loss, reduction in mortality from cardiovascular diseases, diabetes mellitus, and others. The use of ion-ozonated water, which has bactericidal and oxidation-reduction properties, contributes to increasing environmental cleanliness, improving quality, and extending safe storage periods. The authors of [61,62] found that by improving the molecular bonds of gluten proteins under the influence of ozonized water, the stability, stability, and elastic–plastic properties of the dough increase, allowing for the dosage of additives to be increased while maintaining the quality of the finished product. Thus, our research on expanding the range of environmentally friendly products with increased nutritional and biological value through the use of whole-milled flour, ion-ozonated water and carrot powder, aimed at preserving and improving public health, is relevant.

To obtain a reliable assessment of the influence of such factors as the concentration of ion ozone in ion-ozonated water, the drying temperature of pasta, and the content of whole-grain flour and pumpkin powder on the quality indicators of pasta, methods of multifactorial experimental design were used. Data processing and all necessary calculations were carried out using the PLAN sequential regression analysis program [57,58]. This program made it possible to calculate regression coefficients for each quality indicator, check the significance of the regression coefficients and, after removing all insignificant coefficients, determine the necessary statistical characteristics of the resulting regression equations, including checking their adequacy with experimental data.

Maximum protein retention was observed in experiments No. 6, No. 7, and No. 11, along with minimal losses of dry matter in experiments No. 10, No. 11, and No. 12. However, in experiment No. 11, the best results were achieved—a maximum protein content of 14.45% and a minimum loss of dry matter of 5.9%. The optimal values of the influencing factors for this experiment are as follows: x_1_ = 2.5 × 10^−6^ mg/cm^3^, x_2_ = 50 °C, x_3_ = 3.0%, x_4_ = 50 °C.

The calculations have shown that the maximum amount of protein in finished pasta is provided by the following optimal technological regimes:

− pumpkin powder content C_pp_ = 1.0%;
− pasta drying temperature t_d_ = 60 °C.

Under these optimal conditions, the calculated value of the objective function (protein content) is 14.28%. The concentration of ion ozone C_io_ and the temperature of the water for kneading pasta dough do not affect the protein content.

The minimum amount of dry substances transferred into the cooking water is ensured by the following optimal technological regimes:

− concentration of ion ozone in ion-ozonized water C_io_ = 2.5 × 10^−6^ mg/cm^3^;
− pumpkin powder content C_pp_ = 3.0%;
− pasta drying temperature t_d_ = 50 °C.

Under these optimal conditions, the calculated value of the objective function (loss of DM transferred into cooking water) is 5.90%. The water temperature for kneading pasta dough, t_w_, does not affect the protein content.

An analysis of the quality indicators of pasta made from Almaly soft wheat with the addition of pumpkin powder and ion-ozonated water obtained under conditions that provide optimal protein content (y_3_ = 14.28%) and minimal losses of dry matter in cooking water (y_15_ = 5.90%), based on the comparison, showed the following: reducing the dry matter in cooking water to 5.9% stabilizes humidity, acidity, shape retention, and the coefficients of increase in the mass and volume of products, increases the magnitude of total and plastic deformation, reduces protein content by 0.85%, starch by 3.13%, carbohydrates by 4.78%, and increases fat content by 0.76%, fiber by 0.44%, ash by 1.56%, vitamin A by 0.25 mg / 100 g, vitamin E by 0.28 mg/100 g, vitamin C by 0.51 mg/100 g, calcium by 27.4 mg/100 g, magnesium by 39.62 mg/100 g, iron by 1.14 mg/100 g, and zinc by 0.72 mg/100 g.

Data highlight the importance of reducing dry matter to improve nutrient content [63,64]. Based on this, we have selected criteria for optimizing technological regimes for producing pasta using ion-ozonated water and pumpkin powder, reducing the content of dry substances in the cooking water. It has been established that the minimum amount of DM transferred into the cooking water is ensured by the following optimal technological regimes: concentration of ion ozone in ion-ozonized water C_io_ = 2.5 × 10^−6^ mg/cm^3^; pumpkin powder content C_pp_ = 3.0%; pasta drying temperature t_d_ = 50 °C.

As a result of the study, the optimal recipe and modes for preparing pasta made from whole-grain flour, as well as finely dispersed additives from plant materials and ion-ozonated water, were also established. To prepare pasta for 100 kg of soft wheat flour, you need to add 3 kg of pumpkin powder and ion-ozonated water according to the calculation. The water temperature should not exceed 50 °C, the duration of kneading the dough should be no less than 10 min, the kneading pressure should be no less than 5 MPa, the pasta-forming temperature at the exit should be 40–45 °C, the relative air humidity during drying should be 70 ± 2%, the drying temperature should be 50 ± 2 °C, and the drying time at least 6 h.

## 4. Conclusions

Methods involving the multifactorial planning of experiments, using the program of sequential regression analysis (PLAN), allowed us to calculate regression coefficients for each quality indicator, in order to check the significance of regression coefficients. Also, after removing all insignificant coefficients, it allowed us to determine the necessary statistical characteristics of the obtained regression equations, to obtain a reliable assessment of the influence of individual factors (the content of whole-grain flour from soft wheat Almaly, finely dispersed additives from plant materials, and ion-ozonated water) on the quality indicators of pasta. As a result of our research, it was established that in order to reduce the loss of dry matter passing into the cooking water, it is necessary to increase the concentration of ion ozone C_io_, as well as reduce the content of pumpkin powder introduced into pasta C_pp_ and its drying temperature t_d_. Similarly, it has been established that ensuring the minimum amount of dry matter is transferred into the cooking water can be carried out with the following optimal technological regimes: ion-ozone concentration in ion-ozonated water C_io_ = 2.5 × 10^−6^ mg/cm^3^, water temperature t_w_ = 50 °C, pumpkin powder content C_pp_ = 3.0%, pasta drying temperature t_d_ = 50 °C. The development of a range of environmentally friendly products with increased nutritional and biological value due to the introduction of Almaly whole-wheat flour, pumpkin powder, and ion-ozonated water into the recipe for pasta will make it possible to create next-generation products aimed at preserving and improving public health.

## Figures and Tables

**Table 1:** Chemical composition of pumpkin powder and whole-grain flour from Almaly soft wheat varieties.

Name of Indicators	Quantity	
	Pumpkin Powder	Flour from Soft Wheat of the Almaly Variety
humidity, %	4.07	12.66
acidity, %	1.16	2.08
protein content, %	3.55	10.02
starch content, %	0.18	60.27
carbohydrate content %	90.07	68.59
fat content, %	0.82	2.14
fiber content, %	18.94	8.32
ash content, %	4.88	1.48
vitamin A content, mg/100 g	0.01	0
vitamin E content, mg/100 g	0.538	0.58
vitamin C content, mg/100 g	0.639	0
content of Beta carotene, mg/100 g	106.45	0.08
calcium content, mg/100 g	208.19	31.87
potassium content, mg/100 g	4115.61	422.06
magnesium content, mg/100 g	271.37	109.52
iron content, mg/100 g	46.33	1.54
zinc content, mg/100 g	2.94	0.66

**Table 2:** The organoleptic characteristics of the control sample and the studied samples of pasta made from whole-grain flour from Almaly soft wheat and pumpkin powder using ion-ozonated water.

Sample Number	Indicators				
	Surface Condition, %	Shape, %	Color, %	Taste, %	Smell, %
Control sample	80	100	cream, 80	95	100
1	100	100	cream with a yellowish tint, 95	100	100
2	70	100	cream with a yellowish tint, 95	100	100
3	100	100	cream with a yellowish tint, 95	100	100
4	80	100	cream with a yellowish tint, 95	100	100
5	100	100	cream, 80	90	100
6	100	100	cream, 80	90	100
7	100	100	cream, 80	90	100
8	100	100	cream, 80	90	100
9	100	100	light cream with a yellowish tint, 100	100	100
10	100	100	light cream with a yellowish tint, 100	100	100
11	100	100	light cream with a yellowish tint, 100	100	100
12	100	100	light cream with a yellowish tint, 100	100	100
13	100	100	light cream, 90	95	100
14	70	100	light cream, 90	90	100
15	100	100	light cream, 90	90	100
16	100	100	light cream, 90	90	100

**Table 3:** Factors affecting the quality indicators of pasta made from whole-grain flour from Almaly soft wheat and pumpkin powder using ion-ozonated water.

Sample Number	Factors			
	Ion-Ozone Concentration, C_io_ × 10^−6^, mg/cm^3^	Water Temperature, t_w_, °C	Pumpkin Powder Content, C_pp_, %	Drying Temperature, t_d_, °C
	x_1_	x_2_	x_3_	x_4_
1	2.5	60	3.0	60
2	1.5	60	3.0	60
3	2.5	50	3.0	60
4	1.5	50	3.0	60
5	2.5	60	1.0	60
6	1.5	60	1.0	60
7	2.5	50	1.0	60
8	1.5	50	1.0	60
9	2.5	60	3.0	50
10	1.5	60	3.0	50
11	2.5	50	3.0	50
12	1.5	50	3.0	50
13	2.5	60	1.0	50
14	1.5	60	1.0	50
15	2.5	50	1.0	50
16	1.5	50	1.0	50

**Table 4:** The chemical properties of pasta made from whole-grain flour from Almaly soft wheat and pumpkin powder using ion-ozonated water.

Sample Number	Humidity, %	Acidity, Degree	Protein, %	Starch, %	Carbohydrate, %	Fat, %	Fiber, %	Ash, %
	y_1_	y_2_	y_3_	y_4_	y_5_	y_6_	y_7_	y_8_
1	12.6	3.8	12.92	65.76	70.84	3.46	3.58	4.66
2	12.5	3.6	12.96	65.81	70.56	3.35	3.62	4.72
3	12.6	3.8	12.93	65.79	70.37	3.48	3.61	4.76
4	12.4	3.4	12.87	65.10	70.19	3.52	3.71	4.69
5	12.4	4.0	14.21	69.76	75.83	2.61	1.25	2.10
6	12.6	3.6	14.34	68.96	75.78	2.52	1.28	2.07
7	12.4	3.7	14.31	69.54	75.81	2.51	1.27	2.18
8	12.6	3.8	14.27	69.65	75.82	2.56	1.33	2.14
9	13.0	3.4	13.78	65.89	70.12	3.42	2.87	4.31
10	13.0	3.5	12.69	65.93	70.08	3.49	3.78	4.56
11	12.8	3.8	14.45	68.50	75.76	2.55	1.18	3.25
12	12.8	3.6	12.81	65.30	70.25	3.44	3.76	4.78
13	13.0	3.4	13.38	68.74	75.74	2.59	2.33	2.15
14	13.0	3.8	13.43	68.55	75.85	2.63	3.43	3.67
15	13.0	3.7	13.45	68.47	75.94	2.67	1.20	2.17
16	13.0	3.6	13.37	69.47	75.67	2.58	2.38	3.33

**Table 5:** Rheological properties of pasta made from whole-grain flour from Almaly soft wheat and pumpkin powder using ion-ozonated water.

Sample Number	Total Deformation—H_1_, mm	Plastic Deformation—H_2_, mm	Elastic Deformation—H_3_, mm
	y_9_	y_10_	y_11_
1	1.46	0.60	0.90
2	1.26	0.58	0.68
3	1.9	1.08	0.82
4	1.82	0.95	0.87
5	2.32	1.34	0.98
6	3.5	2.20	1.3
7	1.35	0.7	0.73
8	1.99	1.06	1.01
9	2.7	1.55	1.15
10	1.77	1.23	0.97
11	2.54	1.49	0.8
12	2.95	1.72	1.56
13	1.77	0.95	0.96
14	2.59	1.65	1.20
15	2.11	0.77	1.01
16	2.68	1.4	2.08

**Table 6:** Cooking properties of pasta made from whole-grain flour from Almaly soft wheat and pumpkin powder using ion-ozonated water.

Sample Number	Preservation of Form, %	Product Weight Increase Factor (C_m_)	Product Volume Increase Factor (C_v_)	Amount of DM Transferred into the Cooking Water, %	Cooking Time until Done, min.
	y_12_	y_13_	y_14_	y_15_	y_16_
1	92	2.33	1.31	6.15	13
2	84	2.51	1.60	7.0	12
3	96	2.28	1.28	6.09	13
4	88	2.40	1.43	6.90	12
5	97	2.27	1.31	6.08	13
6	97	2.27	1.32	6.05	13
7	97	2.26	1.33	6.18	13
8	98	2.25	1.31	6.14	13
9	100	2.14	1.20	6.0	14
10	100	2.19	1.23	6.0	15
11	100	2.12	1.18	5.9	15
12	100	2.17	1.22	6.0	14
13	97	2.25	1.32	6.1	13
14	84	2.46	1.43	6.85	11
15	97	2.23	1.22	6.05	13
16	97	2.23	1.21	6.02	13

**Table 7:** Content of vitamins, Beta carotene and minerals of pasta made from whole-grain flour from Almaly soft wheat and pumpkin powder using ion-ozonated water.

Sample Number	Vitamin A, mg/100 g	Vitamin E, mg/100 g	Vitamin C, mg/100 g	Beta Carotene, mg/100 g	Ca, mg/100 g	K, mg/100 g	Mg, mg/100 g	Fe, mg/100 g	Zn, mg/100 g
	y_17_	y_18_	y_19_	y_20_	y_21_	y_22_	y_23_	y_24_	y_25_
1	1.44	8.39	4.69	16.56	197.53	493.31	96.26	3.05	1.88
2	1.41	8.43	4.71	16.12	194.54	498.37	89.79	3.10	1.72
3	1.38	8.41	4.56	16.19	187.32	514.09	90.13	3.17	1.63
4	1.40	8.42	4.67	19.37	186.99	523.04	91.12	2.96	1.54
5	0.48	3.79	1.56	5.52	125.47	561.45	49.42	1.87	0.59
6	0.54	3.68	1.54	5.63	123.51	532.68	49.93	1.85	0.61
7	0.38	3.84	1.63	5.74	118.62	545.79	48.97	1.79	0.58
8	0.52	3.57	1.48	7.47	119.41	538.81	51.02	1.78	0.56
9	1.48	8.33	4.85	16.44	194.05	447.18	90.71	2.97	1.52
10	1.46	8.48	4.74	16.38	192.93	452.27	89.12	3.00	1.47
11	0.57	3.57	1.48	5.68	172.98	519.91	81.67	2.01	1.13
12	1.43	8.45	4.65	16.49	176.73	496.42	79.93	3.12	1.32
13	1.17	5.49	3.69	11.13	121.62	560.81	45.23	1.98	1.04
14	1.04	6.64	4.37	14.81	118.18	521.81	50.08	1.81	1.06
15	0.57	3.55	1.59	5.63	117.97	532.63	49.18	1.69	0.61
16	1.07	5.77	2.97	12.71	119.03	527.24	47.93	1.72	0.73

**Table 8:** The regression equations in natural variables and statistical characteristics of the dependencies of the chemical indicators of pasta made from whole-grain flour from Almaly soft wheat and pumpkin powder using ion-ozonated water on the factors influencing them C_io_, t_w_, C_pp_, t_d_.

Regression Equations in Natural Variables	Standard Deviation		Fisher Criterion	
	Experimental	Inadequacy	Settlement	Critical
y_1_ = 15.137 − 0.04375 × t_d_	0.140	0.096	2.13	3.74
y_2_ = 3.656	0.110	0.175	2.53	19.43
y_3_ = 5.551 + 3.481C_pp_ + 0.1569 × t_d_ − 0.06938 × C_pp_ × t_d_	0.180	0.420	5.43	19.41
y_4_ = 70.709 − 1.566 × C_pp_	0.580	0.828	2.04	19.42
y_5_ = 78.197 − 2.392 × C_pp_	0.680	1.367	4.04	19.42
y_6_ = 2.206 + 0.3775 × C_pp_	0.140	0.231	2.73	19.42
y_7_ = −8.643 × C_io_ × 10^−6^ + 0.5316 × t_w_ − 4.250 × C_pp_ + 0.1435 × C_io_ × 10^−6^ × t_d_ − 0.00881 × t_w_ × t_d_ + 0.0905 × C_pp_ × t_d_	0.110	0.306	7.72	19.39
y_8_ = 22.225 − 6.790 × C_io_ × 10^−6^ − 2.277 × C_pp_ − 0.3572 × t_d_ + 0.1135 × C_io_ × 10^−6^ × t_d_ + 0.0595 × C_pp_ × t_d_	0.160	0.275	2.96	19.39

**Table 9:** The regression equations in natural variables and statistical characteristics of the dependencies of rheological properties of pasta made from whole-grain flour from Almaly soft wheat and pumpkin powder using ion-ozonated water on the factors influencing them C_io_, t_w_, C_pp_, t_d_.

Regression Equations in Natural Variables	Standard Deviation		Fisher Criterion	
	Experimental	Inadequacy	Settlement	Critical
y_9_ = 19.116 − 1.304 × C_io_ × 10^−6^ − 0.3007 × t_w_ + 4.103 × C_pp_ − 0.3585 × t_d_ − 0.5012 × C_io_ × 10^−6^ × C_pp_ − 0.05087 × t_w_ × C_pp_ + 0.00732 × t_w_ × t_d_ − 0.0441 × C_pp_ × t_d_	0.102	0.395	15.01	19.35
y_10_ = −0.9699 × C_io_ × 10^−6^ + 4.061 × C_pp_ − 0.0409 × t_d_ + 0.3422C_o_ × 10^−6^ × C_pp_ − 0.04409 × t_w_ × C_pp_ + 0.00182 × t_w_ × t_d_ −0.04317 × C_pp_ × t_d_	0.053	0.196	13.63	19.38
y_11_ = 26.385 − 5.422 × C_io_ × 10^−6^ − 0.3292 × t_w_− 0.4700 × C_pp_ − 0.3235 × t_d_ + 0.0500 × C_io_ × 10^−6^ × t_w_ + 0.1875 × C_o_ × 10^−6^ × C_pp_ + 0.03650 × C_io_ × 10^−6^ × t_d_ + 0.00400 × t_w_ × t_d_	0.049	0.198	16.31	19.35

**Table 10:** The regression equations in natural variables and statistical characteristics of the dependencies of cooking properties of pasta made from whole-grain flour from Almaly soft wheat and pumpkin powder using ion-ozonated water on the factors influencing them C_io_, t_w_, C_pp_, t_d_.

Regression Equations in Natural Variables	Standard Deviation		Fisher Criterion	
	Experimental	Inadequacy	Settlement	Critical
y_12_ = 52.625 × C_pp_ + 1.735 × t_c_ − 0.959 × C_pp_ × t_d_	0.110	0.175	2.53	19.43
y_13_ = 2.272	0.061	0.108	3.12	19.43
y_14_ = 1.306 − 0.338 × C_pp_ + 0.00615 × C_pp_ × t_d_	0.046	0.088	3.64	19.42
y_15_ = 6.112 − 0.177 × C_io_ × 10^−6^ × C_pp_ + 0.00742 × C_pp_ × t_d_	0.150	0.290	3.74	19.42
y_16_ = 8.350 × C_pp_ + 0.225 × t_d_ − 0.145 × C_pp_ × t_d_	0.340	0.632	3.46	19.42

**Table 11:** The regression equations in natural variables and statistical characteristics of the dependencies of the content of vitamins, Beta carotene and minerals of pasta made from whole-grain flour from Almaly soft wheat and pumpkin powder using ion-ozonated water on the factors influencing them C_io_, t_w_, C_pp_, t_d_.

Regression Equations in Natural Variables	Standard Deviation		Fisher Criterion	
	Experimental	Inadequacy	Settlement	Critical
y_17_ = 4.112 − 3.986 × C_io_ × 10^−6^ + 0.07857 × t_w_ − 1.5396 × C_pp_ + 0.0410 × C_io_ × 10^−6^ × t_w_ + 0.02829 × C_io_ × 10^−6^ × t_d_ −0.00253 × t_w_ × t_d_ + 0.03345 × C_pp_ × t_d_	0.045	0.152	11.38	19.37
y_18_ = 15.092 − 21.640 × C_io_ × 10^−6^ + 0.6019 × t_w_ − 6.425 × C_pp_ + 0.1402 × t_w_ + 0.2349 × C_io_ × 10^−6^ × t_d_ −0.01430 × t_w_ × t_d_+0.1465 × C_pp_ × t_d_	0.280	0.792	8.01	19.37
y_19_ = −14.502 × C_io_ × 10^−6^ + 0.6155 × t_w_ − 5.6075 × C_pp_ + 0.1826 × t_d_ + 0.1108 × C_io_ × 10^−6^ × t_w_ + 0.1413 × C_io_ × 10^−6^ × t_d_ − − 0.01363 × t_w_ × t_d_ + 0.1196 × C_pp_ × t_d_	0.140	0.517	13.64	19.37
y_20_ = −55.624 × C_io_ × 10^−6^ + 2.026 × t_w_ − 19.680 × C_pp_ + 1.066 × t_d_ + 0.508 × C_io_ × 10^−6^ × t_w_ + 0.4437 × C_io_ × 10^−6^ × t_d_ − 0.05234 × t_w_ × t_d_ + 0.4198 × C_pp_ × t_d_	0.520	1.338	6.62	19.37
y_21_ = 86.77 + 33.704 × C_pp_	6.540	6.558	1.01	19.42
y_22_ = 464.163 + 1.866 × t_d_ − 0.4561 × t_w_ × C_pp_	7.830	18.374	5.51	19.42
y_23_ = 29.159 + 19.811 × C_pp_	3.340	3.949	1.40	19.42
y_24_ = 0.6018 × C_io_ × 10^−6^ + 1.196 × C_pp_ − 0.3100 × C_io_ × 10^−6^ × C_pp_	0.118	0.287	5.92	19.42
y_25_ = 3.511 − 0.6128 × C_io_ × 10^−6^ − 1.2687 × C_pp_ − 0.05788 × t_d_ + 0.01107 × C_io_ × 10^−6^ × t_w_ + 0.03038 × C_pp_ × t_d_	0.051	0.200	3.84	19.39

**Table 12:** Values of quality indicators for pasta made from Almaly soft wheat, finely divided pumpkin powder and ion-ozonated water obtained under conditions that provide optimal protein content (y_11_) and minimal losses of dry substances in cooking water (y_6_).

Quality Indicators		Min		Opt			Max
				y_11_	y_6_		
y_1_	–moisture content of pasta, %	12.0	≤	12.51	12.95	≤	14.0
y_2_	–acidity of pasta, degrees	3.0	≤	3.66	3.66	≤	4.5
y_3_	–protein content, %	12.55	≤	14.28	13.43	≤	14.6
y_4_	–starch content, %	65.0	≤	69.14	66.01	≤	69.9
y_5_	–carbohydrate content, %	70.0	≤	75.80	71.02	≤	76.0
y_6_	–fat content, %	2.45	≤	2.58	3.34	≤	3.6
y_7_	–fiber content, %	1.1	≤	1.27	1.71	≤	3.8
y_8_	–ash content, %	2.0	≤	2.11	3.67	≤	4.8
y_9_	–complete deformation H_1,_ mm	1.0	≤	2.25	3.0	≤	3.0
y_10_	–plastic deformation H_2,_ mm	0.4	≤	1.33	1.74	≤	2.25
y_11_	–elastic deformation H_3,_ mm	0.58	≤	1.22	1.00	≤	2.2
y_12_	–preservation of the shape of pasta, %	82.0	≤	99.19	100.0	≤	100.0
y_13_	–coefficient of increase in mass of products (C_m_)	2.1	≤	2.27	2.27	≤	2.55
y_14_	–coefficient of increase in the volume of products (C_o_)	1.19	≤	1.34	1.21	≤	1.63
y_15_	–amount of dry matter transferred into the cooking water, %	5.8	≤	6.29	5.90	≤	7.2
y_16_	–cooking time until done, min	10.0	≤	13.14	14.55	≤	15.5
y_17_	–vitamin A content, mg/100 g	0.3	≤	0.55	0.80	≤	1.5
y_18_	–vitamin E content, mg/100 g	3.45	≤	3.64	4.92	≤	8.55
y_19_	–vitamin C content, mg/100 g	1.4	≤	1.68	2.19	≤	4.9
y_20_	–Beta carotene content, mg/100 g	5.4	≤	8.34	7.59	≤	19.5
y_21_	–calcium content, mg/100 g	117.85	≤	120.48	187.88	≤	197.6
y_22_	–potassium content, mg/100 g	447.0	≤	553.31	489.04	≤	561.5
y_23_	–magnesium content, mg/100 g	45.1	≤	48.97	88.59	≤	96.35
y_24_	–iron content, mg/100 g	1.65	≤	1.63	2.77	≤	3.25
y_25_	–zinc content, mg/100 g	0.5	≤	0.50	1.22	≤	1.95

## Data Availability

The original contributions presented in the study are included in the article; further inquiries can be directed to the corresponding author.

## References

[1] Kane A., Bèye N.F., Sow A., Diakhaté P.A., Ndiaye N.Y., Cissé M. (2023). Profilef the Functionalategoriesf Food Additives in Industrial Foods Marketed in Senegal. Food Nutr. Sci..

[2] Kane A., Mbodji H., Sylla P.M.D.D., Sow A., Tamba A., Mbengue M., Cissé M. (2024). Consumers’ Perception and Knowledge of Food Additives in Senegal: A Pilot Study. Open J. Appl. Sci..

[3] Ivashchenko A.A. (2009). Flora of Kazakhstan.

[4] Baytenov M.S. (1999). Flora of Kazakhstan: Illustrated Guide to Families and Genera.

[5] Polievoda Y., Revva V., Tverdokhlib I. (2023). Features of the grain micronization process. Vib. Eng. Technol..

[6] Sots S., Kustov I., Donii O. (2024). Authentic wheat grain is the raw material for modern cereal products. Grain Prod. Mix. Fodder’s.

[7] Naumenko N., Potoroko I., Kalinina I., Fatkullin R., Ivanisova E. (2021). The Influence of the Use of Whole Grain Flour from Sprouted Wheat Grain on the Rheological and Microstructural Properties of Dough and Bread. Int. J. Food Sci..

[8] van der Kamp J.-W., Jones J.M., Miller K.B., Ross A.B., Seal C.J., Tan B., Beck E.J. (2021). Consensus, Global Definitions of Whole Grain as a Food Ingredient and of Whole-Grain Foods Presented on Behalf of the Whole Grain Initiative. Nutrients.

[9] Ye E.Q., Chacko S.A., Chou E.L., Kugizaki M., Liu S. (2013). Greater whole-grain intake is associated with lower risk of type 2 diabetes, cardiovascular disease, and weight gain. J. Nutr..

[10] Singh A.K., Rehal J., Kaur A., Jyot G. (2013). Enhancement of Attributes of Cereals by Germination and Fermentation: A Review. Crit. Rev. Food Sci. Nutr..

[11] Giacco R., Della Pepa G., Luongo D., Riccardi G. (2011). Whole grain intake in relation to body weight: From epidemiological evidence to clinical trials. Nutr. Metab. Cardiovasc. Dis..

[12] Priebe M., van Binsbergen J., de Vos R., Vonk R.J. (2008). Whole grain foods for the prevention of type 2 diabetes mellitus. Cochrane Database Syst. Rev..

[13] Arnlov J., Sundstrom J., Ingelsson E., Lind L. (2010). Impact of BMI and the Metabolic Syndrome on the Risk of Diabetes in Middle-Aged Men. Diabetes Care.

[14] Harris K.A., Kris-Etherton P.M. (2010). Effects of Whole Grains on Coronary Heart Disease Risk. Curr. Atheroscler. Rep..

[15] Yadav D.N., Sharma M., Chikara N., Anand T., Bansal S. (2014). Quality Characteristics of Vegetable-Blended Wheat–Pearl Millet Composite Pasta. Agric. Res..

[16] Naji-Tabasi S., Niazmand R., Modiri-Dovom A. (2021). Application of mucilaginous seeds (*Alyssum homolocarpum* and *Salvia macrosiphon Boiss*) and wheat bran in improving technological and nutritional properties of pasta. J. Food Sci..

[17] Sicignano A., Di Monaco R., Masi P., Cavella S. (2015). From raw material to dish: Pasta quality step by step. J. Sci. Food Agric..

[18] Gałkowska D., Witczak T., Witczak M. (2021). Ancient Wheat and Quinoa Flours as Ingredients for Pasta Dough—Evaluation of Thermal and Rheological Properties. Molecules.

[19] Lux (née Bantleon) T., Spillmann F., Reimold F., Erdös A., Lochny A., Flöter E. (2023). Physical quality of gluten-free doughs and fresh pasta made of amaranth. Food Sci. Nutr..

[20] Zavalishina O.M., Kuznetsova T.A., Korneeva A.V. (2021). The quality of pasta products made from various kinds of flour. IOP Conf. Ser. Earth Environ. Sci..

[21] Wójtowicz A., Oniszczuk A., Kasprzak K., Olech M., Mitrus M., Oniszczuk T. (2020). Chemical composition and selected quality characteristics of new types of precooked wheat and spelt pasta products. Food Chem..

[22] Rodrigues D.S., Cavalcanti M.T., Gomes C.A., Araújo J.S., Lima R.P., Moreira I.D.S., Monteiro S.S., Pereira E.M. (2023). Partial Substitution of Wheat Flour with Palm Flour in Pasta Preparation. Appl. Sci..

[23] Fois S., Piu P.P., Sanna M., Roggio T., Catzeddu P. (2018). Starch digestibility and properties of fresh pasta made with semolina-based liquid sourdough. LWT.

[24] Catzeddu P., Fois S., Tolu V., Sanna M., Braca A., Vitangeli I., Anedda R., Roggio T. (2023). Quality Evaluation of Fresh Pasta Fortified with Sourdough Containing Wheat Germ and Wholemeal Semolina. Foods.

[25] Karimova G., Niyazbekova R., Al Azzam K., Mashanova N., Negim E.-S., Ibzhanova A. (2023). Development of new technologies (recipes) to produce pasta with the addition of millet and the determination of organoleptic and physicochemical quality indicators. Potravin. Slovak J. Food Sci..

[26] Karimova G., Niyazbekova R., Al Azzam K., Negim E.-S. (2024). Quality characteristics of the shape preservation of cooked pasta with millet addition at different time intervals. Potravin. Slovak J. Food Sci..

[27] Dhiraj B., Prabhasankar P. (2013). Influence of Wheat-Milled Products and Their Additive Blends on Pasta Dough Rheological, Microstructure, and Product Quality Characteristics. Int. J. Food Sci..

[28] Peressini D., Tat L., Sensidoni A. (2018). Performance comparison between different hydrocolloids to improve quality of pasta made from common wheat. Eur. Food Res. Technol..

[29] Eliseeva L.G. (2021). The benefits of pumpkin-Top 7 useful properties and interesting facts. J. Healthy Nutr. Diet..

[30] de Lira R.P., da Silva T.I., Sales G.N.B., da Silva K.G., dos Santos Formiga A., dos Santos K.P., de Sousa F.F., da Costa Silva I., de Queiroga R.C.F., de Almeida F.A. (2024). Impact of Harvest Time and Storage on the Quality and Bioactive Compounds of ‘Brasileirinha’ Pumpkin. J. Plant Growth Regul..

[31] Minarovičová L., Lauková M., Kohajdová Z., Karovičová J., Kuchtová V. (2017). Effect of pumpkin powder incorporation on cooking and sensory parameters of pasta. Potravin. Slovak J. Food Sci..

[32] Espinosa-Solis V., Zamudio-Flores P.B., Tirado-Gallegos J.M., Ramírez-Mancinas S., Olivas-Orozco G.I., Espino-Díaz M., Hernández-González M., García-Cano V.G., Sánchez-Ortíz O., Buenrostro-Figueroa J.J. (2019). Evaluation of Cooking Quality, Nutritional and Texture Characteristics of Pasta Added with Oat Bran and Apple Flour. Foods.

[33] Iztayev A., Kulazhanov T., Iskakova G., Alimardanova M., Zhienbaeva S., Iztayev B., Tursunbayeva S., Yakiyayeva M. (2023). The innovative technology of dough preparation for bread by the accelerated ion–ozone cavitation method. Sci. Rep..

[34] Iztayev A., Tursunbayeva S., Zhiyenbayeva S., Iskakova G., Matibayeva A., Izteliyeva R., Yakiyayeva M. (2023). Highly Efficient Technology for Making Bread Using an Ion-ozone Mixture. Int. J. Technol..

[35] Tursunbayeva S., Iztayev A., Mynbayeva A., Alimardanova M., Iztayev B., Yakiyayeva M. (2021). Development of a highly efficient ion-ozone cavitation technology for accelerated bread production. Sci. Rep..

[36] Iztayev A., Kulazhanov T.K., Yakiyayeva M.A., Zhakatayeva A.N., Baibatyrov T.A. (2021). Method for the safe storage of sugar beets using an ion-ozone mixture. Acta Sci. Pol. Technol. Aliment..

[37] Iztayev B., Yakiyayeva M., Magomedov M., Iztayev A., Kenzhekhojayev M., Spandiyarov Y. (2021). Devising technology of the accelerated method for making yeast-free bakery products from wheat flour. East.-Eur. J. Enterp. Technol..

[38] Iztayev A., Urazaliev R., Yakiyayeva M., Maemerov M., Shaimerdenova D., Iztayev B., Toxanbayeva B., Dauletkeldi Y. (2018). Regress models of ion-ozon treatment without and with cavitation, describing changes of indicators for grain crops quality. Acta Tech. CSAV (Ceskoslovensk Akad. Ved).

[39] Shingala A.M., Dabhi M.N., Joshi N.U. (2024). Ozone-based grain storage: A green technology with great potential for improving food safety. Futur. Trends Agric. Eng. Food Sci..

[40] Bai Y.-P., Zhou H.-M. (2021). Impact of aqueous ozone mixing on microbiological, quality and physicochemical characteristics of semi-dried buckwheat noodles. Food Chem..

[41] Burak L. (2022). Using ozonizing technology in the food industry. Zenodo Sci. Eur..

[42] Guo X., Jiang Y., Xing J., Zhu K. (2020). Effect of ozonated water on physicochemical, microbiological, and textural properties of semi-dried noodles. J. Food Process. Preserv..

[43] (2010). Pasta Products. General Specifications.

[44] Methodology for Organoleptic Quality Assessment of Pasta Products of Group B. Internet Resource. https://studwood.net/2001645/marketing/metodika_osuschestvleniya_organolepticheskoy_otsenki_kachestva_makaronnyh_izdeliy_gruppy?ysclid=m235cqblpi976226772.

[45] (2014). Pasta Products. Acceptance Rules and Methods for Determining Quality.

[46] Medvedev G.M. (2006). Pasta technology. Part 3. Textbook for universities. 3 Parts.

[47] (2009). Grain and Products of Its Processing. Protein Determination Method.

[48] (1992). Grain and Products of Its Processing. Fat Determination Method.

[49] (2009). Grain and Products of Its Processing. Method for Determination of Starch.

[50] (2020). Methods for Determining Crude Fiber Content Using Intermediate Filtration.

[51] (2019). Functional Food Products. Method for Determining Vitamin A.

[52] (2017). Food Products. Determination of Vitamin C Using High Performance Liquid Chromatography.

[53] (2015). Food Products. Determination of Vitamin E Content (Alpha-, Beta-, Gamma- and Delta-Tocopherols) by High-Performance Liquid Chromatography.

[54] (2016). Food Products. Determination of Vitamin A Content by High-Performance Liquid Chromatography. Part 2: Measuring Beta-Carotene Content.

[55] (2020). Food Products. Determination of Vitamin A Content by High Performance Liquid Chromatography. Part 1: Measurement of Total E-Retinol and 13-Z-Retinol.

[56] (2014). Feed, Compound Feed. Determination of Calcium, Copper, Iron, Magnesium, Manganese, Potassium, Sodium and Zinc Content by Atomic Absorption Spectrometry.

[57] Ostapchuk N.V. (1991). Tutorial. Fundamentals of Mathematical Modeling of Food Production Processes.

[58] Ostapchuk M.V., Stankevich H.M. (2006). Mathematical Modeling on a Computer: Textbook.

[59] Huang T., Xu M., Lee A., Cho S., Qi L. (2015). Consumption of whole grains and cereal fiber and total and cause-specific mortality: Prospective analysis of 367,442 individuals. BMC Med..

[60] Johnsen N.F., Frederiksen K., Christensen J., Skeie G., Lund E., Landberg R., Johansson I., Nilsson L.M., Halkjær J., Olsen A. (2015). Whole-grain products and whole-grain types are associated with lower all-cause and cause-specific mortality in the Scandinavian HELGA cohort. Br. J. Nutr..

[61] Kulazhanov K.S., Iztayev A.I., Iskakova G.K. (2008). Improving Bread Technology Based on Leguminous Flour and Ozonated Water: Monograph.

[62] Akkozha I.S., Iztayev A., Iztayev B.A., Mukhtarkhanova R.B., Yakiyayeva M.A. (2023). Accelerated technology for bread preparation using activated water. Potravin. Slovak J. Food Sci..

[63] Medvedev P.V., Fedotov V.A. (2016). Methods for assessing the pasta qualities of wheat grain. Int. Res. J..

[64] Malyutina T.N., Turenko V.Y. (2016). Study the effect of non-traditional type of flour on the quality of pasta products made of soft wheat. Proc. Voronezh State Univ. Eng. Technol..

